# CXCR3 inhibitors for therapeutic interventions: current status and perspectives

**DOI:** 10.3389/fphar.2025.1556196

**Published:** 2025-07-25

**Authors:** Rongrong Huo, Yu Jiang, Li Zhang, Shufang Du, Dan Zhou

**Affiliations:** ^1^ State Key Laboratory of Oral Diseases, West China School of Stomatology, Sichuan University, Chengdu, Sichuan, China; ^2^ West China Hospital, Sichuan University, Chengdu, Sichuan, China

**Keywords:** CXCR3, tumor, inflammatory diseases,, CXCR3 inhibitors, molecule antagonists,, future clinical treatments

## Abstract

CXC chemokine receptor 3 (CXCR3) is a G protein-coupled chemokine receptor that plays a key role in regulating immune responses and is involved in various pathological processes, particularly in tumor development and inflammatory diseases, making it a novel target for clinical therapy. The expression of CXCR3 and its ligands—CXCL9, CXCL10, CXCL11, CXCL4, and CXCL4L1—is closely associated with the onset and progression of numerous diseases. With a deeper understanding of the mechanisms underlying CXCR3 function, significant progress has been made in the development of small molecule antagonists targeting CXCR3, some of which have entered clinical trials and demonstrated therapeutic potential. This review provides an overview of the structure and signaling pathways of CXCR3, its biological functions in cancer and inflammatory diseases, and highlights the innovative roles of CXCR3 in these diseases. Furthermore, it discusses recent advances in the development of small molecule antagonists, particularly those that have been tested in clinical settings, such as AMG 487 and ACT-777991. These studies provide a scientific foundation for the development of novel CXCR3 antagonists and may offer new directions for future clinical treatments.

## 1 Introduction

CXCR3 is a G protein-coupled chemokine receptor, belonging to the CXCR subfamily (CXCR1–CXCR7) of the G protein-coupled receptor (GPCR) family. It is also referred to as G protein-coupled receptor 9 (GPR9) and is designated as CD183. Since its initial discovery, CXCR3 has been shown to play a crucial role in a wide range of physiological and pathological processes. CXCR3 antagonists were initially proposed for the treatment of inflammatory and autoimmune diseases; however, recent studies suggest that they may have therapeutic potential in a broader spectrum of diseases (Wijtmans et al., 2014; Wijtmans et al., 2011; Wijtmans et al., 2008).

Chemokines are a class of low molecular weight soluble cytokines, typically ranging from 8 to 15 kDa, that exert biological effects through their interaction with G protein-coupled receptors (GPCRs). GPCRs are one of the key targets in drug development. Chemokines play essential roles in various biological processes, including development, angiogenesis, inflammatory responses, immune diseases, and cancer (Andrews and Cox, 2016).

The CXC chemokine family is an important branch of the chemokine family, characterized by the presence of an “X” amino acid located between cysteine residues at the amino terminus. CXC chemokines are involved in various biological processes, including cell chemotaxis, regulation of cell growth, induction of apoptosis, and modulation of anti-angiogenic activity (Billottet et al., 2013). CXCL9/MIG, CXCL10/IP-10, CXCL11/I-TAC, CXCL4/PF-4, and CXCL4L1/PF4V1 are all members of the CXC chemokine family, and they exert biological effects by binding to the CXCR3 receptor.

The gene encoding CXCR3 (Gene ID: 2833) is located on the X chromosome (Q13). This receptor is expressed on activated T cells, NK cells, mast cells, and dendritic cells. Its expression is not limited to leukocytes but also includes fibroblasts, endothelial cells, and tumor cells.

CXCR3 has been recognized to play a critical role in several human diseases, with the expression of CXCR3 and its ligands associated with various inflammatory diseases (Qin et al., 1998), tumors (Ma et al., 2015), transplant rejection (Sørensen et al., 1999), and others. Three subtypes of CXCR3 have been identified (CXCR3-A, CXCR3-B, and CXCR3-alt), with the two major subtypes, CXCR3-A and CXCR3-B, believed to induce opposing physiological functions (Va et al., 2015). CXCR3-A, primarily found on hematopoietic cells, appears to mediate tumor progression signals by promoting cell proliferation, survival, chemotaxis, invasion, and metastasis. In contrast, CXCR3-B, mainly expressed on endothelial cells, seems to mediate tumor suppression signals by promoting growth inhibition, apoptosis, and vascular regression (Ma et al., 2015). Therefore, the dysregulated expression of CXCR3-A and CXCR3-B may influence tumor progression.

The development of drugs targeting chemokine receptors has always been a challenge. Although G protein-coupled receptors (GPCRs) are popular drug targets, very few chemokine receptor antagonists have reached the market, aside from HIV entry inhibitors. This may be due to the relatively late discovery of chemokine receptors and the high redundancy within the chemokine system, which complicates the prediction of the therapeutic efficacy of chemokine receptor antagonists. Nonetheless, the upregulated expression of CXCR3 and its ligands in a variety of diseases has been associated with several inflammatory conditions, including multiple sclerosis, rheumatoid arthritis, atherosclerosis, chronic obstructive pulmonary disease, inflammatory bowel disease, inflammatory skin disorders such as psoriasis, as well as hepatitis C infection in the liver, sarcoidosis, and SARS. Additionally, CXCR3 plays a key role in transplant rejection, metastasis of melanoma and colon cancer, and may act as a co-receptor for certain HIV strains. To explore the therapeutic potential of the CXCR3 receptor system, various preclinical approaches have been employed, including: 1) generating CXCR3 knockout mice; 2) using antibodies targeting CXCR3 or its endogenous ligands; 3) inhibiting CXCR3 with protein-based antagonists; and 4) targeting CXCR3 with small molecule drugs. These studies have provided valuable insights for the further development of CXCR3 antagonists.

Due to the critical role of CXCR3 and its ligands in various diseases, CXCR3 has emerged as a promising target for anti-tumor and anti-inflammatory therapies. CXCR3 antagonists can block the binding of CXCR3 to its ligands, thereby inhibiting the biological signaling axis, which may offer therapeutic benefits for conditions such as tumors and autoimmune diseases. In recent years, many research institutions have focused on developing CXCR3 antagonists. Among them, Chemocentryx has been particularly prominent, identifying a series of dihydro-1,2,4-triazolo[3,4-b]quinolin-3-yl derivatives, including potent and effective CXCR3 antagonists (e.g., AMG487). In preclinical studies, AMG487 was shown to block immune cell migration and exhibited excellent potency, high selectivity, and good oral bioavailability. Unfortunately, this inhibitor failed to demonstrate any signs of efficacy in Phase II clinical trials, and the trial was terminated. In the latest research, ACT-777991, a novel CXCR3 antagonist, has undergone preliminary human studies in healthy adults to evaluate its pharmacokinetics, pharmacodynamics, and safety. The study demonstrated that ACT-777991 was well-tolerated at both single and multiple escalating doses and exhibited pharmacokinetic and pharmacodynamic properties suitable for further clinical development. Moreover, ACT-777991, in combination with an anti-CD3 antibody, showed synergistic effects in experimental models, increasing the sustained remission rate in a type 1 diabetes model.

Although CXCR3 antagonists have demonstrated therapeutic efficacy in animal models of disease, studies validating the effectiveness of these models in humans are still lacking. Furthermore, more in-depth research is needed on the pharmacokinetic properties, dose-dependency, signaling bias, and potential side effects of these drugs.

## 2 Structures, subtypes, and ligands of CXCR3

The precise structure of a protein is crucial for understanding its interactions with various molecules and may facilitate the discovery of new ligands with better affinity, selectivity, or safety profiles.

### 2.1 Structure of CXCR3

**FIGURE 1:**
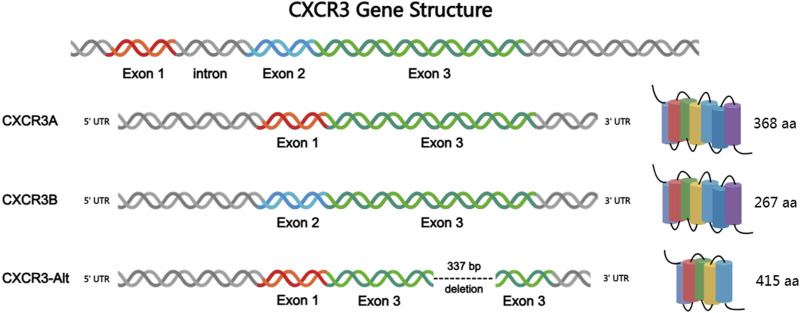
Structures of CXCR3 gene and three subtypes.

As shown in Figure 1, CXCR3 is a typical seven-transmembrane G protein-coupled receptor, featuring an external N-terminus, three extracellular loops (ECL1-3), three intracellular loops (ICL1-3), and an intracellular C-terminus. Recent structural biology studies have revealed that the second extracellular loop (ECL2) of CXCR3 plays a key role in receptor activation, while the N-terminus is involved in initial ligand recognition (Jiao et al., 2024; Xanthou et al., 2003). CXCL11 promotes receptor activation by interacting with the N-terminus and ECL2 of CXCR3 through its N-terminus (Jiao et al., 2024). Additionally, small-molecule agonists, such as VUF10661 and VUF11418, activate CXCR3 through distinct binding modes, offering new insights for drug design (Jiao et al., 2024; Saha et al., 2025).

### 2.2 Three subtypes of CXCR3

Based on the composition of the receptor’s N-terminal, CXCR3 can be classified into three subtypes (Figure 1): CXCR3-A, CXCR3-B, and the truncated variant CXCR3-alt (Singh et al., 2013). Among these, CXCR3-A and CXCR3-B are the most extensively studied isoforms. CXCR3-A is primarily expressed on activated T lymphocytes and natural killer (NK) cells, whereas CXCR3-B is predominantly found on endothelial cells. The research on CXCR3-alt is relatively limited, and it is currently believed to mainly exert its biological effects in conjunction with interferon-inducible T cell alpha chemoattractant (I-TAC) (Billottet et al., 2013). Both CXCR3-A and CXCR3-B encode seven transmembrane domains, and both proteins are coupled to heterotrimeric G proteins (HTGPs), mediating their downstream signaling. The number of CXCR3-alt transmembrane helices is predicted to be 4 to 5 (Ehlert et al., 2004). CXCR3-A consists of 368 amino acids and is encoded by two exons separated by an intron, coupling with a Gai protein. The coding sequence of CXCR3-A includes three amino acids from exon 1, with the remainder from exon 2. CXCR3-B, composed of 415 amino acids, results from selective splicing at the 5′end of the second exon. The coding sequence of CXCR3-B comes from exon 2 and a retained intronic sequence that is adjacent to the 5′end of exon 2. Furthermore, the inserted sequence contains a selective start codon, leading to the loss of the four N-terminal residues encoded by the first exon, which are replaced by a new 51 amino acid N-terminal tail. In terms of sequence, the only difference between CXCR3-A and CXCR3-B lies in their N-terminal regions. Specifically, CXCR3-B has an additional 48 amino acids at its N-terminus, which are absent in CXCR3-A. Despite their sequence similarities, the behavior of CXCR3-A and CXCR3-B is quite distinct. Activation of CXCR3-A has been shown to induce chemotaxis and proliferation in various cell types, increase intracellular calcium levels, and enhance cell survival upon overexpression. In contrast, activation of CXCR3-B inhibits migration and proliferation, and induces apoptosis. Overexpression of CXCR3-B significantly reduces DNA synthesis and upregulates the apoptosis-associated HMEC-1 death signaling pathway.

CXCR3 subtypes exhibit distinct organ-specific distribution patterns. Study (Boyé et al., 2017) showed that CXCR3-A is predominantly enriched in CD8^+^ tissue-resident memory T cells (Trm) and tumor-infiltrating NK cells, accounting for up to 68% of portal vein-associated lymphocytes in the liver. CXCR3-B is specifically expressed in liver sinusoidal endothelial cells (with expression levels 3.2 times higher than CXCR3-A) and glomerular mesangial cells. Recent cryo-electron microscopy studies have revealed that the N-terminal extension of CXCR3-B forms β-sheets to interact with laminin in the hepatic extracellular matrix, which may explain its high expression in the liver. CXCR3-alt is expressed in choroid plexus epithelial cells at levels over five times higher than other subtypes, and its truncated structure may be involved in blood-brain barrier regulation.

### 2.3 Ligands of CXCR3

As shown in Table 1 the ligands of CXCR3 are mainly divided into two categories: IFN-γ-induced ligands CXCL9, CXCL10, and CXCL11, and platelet-derived ligands CXCL4 and CXCL4L1. CXCL9, CXCL10, and CXCL11 are regulated by different promoters, exhibit distinct expression patterns, and bind to CXCR3-A and CXCR3-B with different affinities (Karin, 2020). CXCR3-A has high affinity for CXCL9, CXCL10, and CXCL11, while CXCR3-B has a higher affinity for CXCL4 and CXCL10. CXCL4 does not activate CXCR3-A, but CXCL4L1 binds with moderate affinity to both CXCR3-A and CXCR3-B. Unlike the IFN-γ-induced ligands, CXCL4 and CXCL4L1 are not induced by IFN-γ, but are released by activated platelets. CXCL4L1 shares high sequence similarity with CXCL4, but subtle structural differences result in distinct functions, such as differences in their potential to inhibit angiogenesis (Zhou et al., 2019).

**TABLE 1:** Detailed information on CXCR3 ligands.

	CXCL4	CXCL4L1	CXCL9	CXCL10	CXCL11
Gene(HGNC)	NM_002619	NM_002620	NM_002416	NM_001565	NM_005409
	4q13.3	4q13.3	4q21.1	4q21.1	4q21.1
Mature protein	70 AA	70 AA	103 AA	77 AA	73 AA
Producer cells	Platelets Monocytes T cells pDC	Platelets Smooth muscle cells	HMVEC Fibroblasts Macrophages PBMC	Fibroblasts Monocytes T cells Keratinocytes	Fibroblasts Astrocytes PBMC
Inducers	Thrombin (platelets) Phorbol ester (T cells)	Thrombin (platelets)	IFN-γ	TNF-α (fibroblasts) IFN-α,IFN-β,IFN-γ	TNF-α (fibroblasts) IFN-γ,IFN-β
Processing					
-Protease	N.D.	N.D.	DPPIV/CD26	DPPIV/CD26	DPPIV/CD26
-Peptide product	CXCL4^17−70^	N.D.	CXCL9^3−103^	CXCL10^3−77^	CXCL11^3−73^
-Effect on					
Chemotaxis	N.D.		↓	↓	↓
Angiostasis	↑		=	=	=

### 2.4 Oligomerization of CXCR3

CXCR3 exhibits dynamic oligomerization properties that critically regulate its signaling bias and cellular trafficking. As a GPCR, CXCR3 forms homo-oligomers (CXCR3-A/CXCR3-A, CXCR3-B/CXCR3-B) and hetero-oligomers with other chemokine receptors such as CXCR4 and CCR5 (Breitwieser, 2004; Watts et al., 2013). Structural studies reveal that CXCR3-A homo-oligomerization occurs via transmembrane domain (TM) interactions (TM1, TM4, TM5), stabilizing active conformations essential for Gαi coupling and cAMP/PKC pathway activation (Boyé et al., 2017). In contrast, CXCR3-B oligomerization is modulated by its extended N-terminal domain, which restricts surface expression while enhancing β-arrestin recruitment and ERK phosphorylation (D’Uonnolo et al., 2022). Hetero-oligomerization with CXCR4 induces negative binding cooperativity—CXCL12 binding to CXCR4 suppresses CXCL11-mediated CXCR3 activation, a mechanism implicated in tumor immune evasion (Watts et al., 2013). Notably, CXCR3-Alt acts as a dominant-negative variant by forming nonfunctional oligomers with CXCR3-A/B, effectively reducing their membrane localization (Nguyen et al., 2023). These oligomeric states directly influence ligand selectivity; for example, CXCR3-CXCR4 heterodimers redirect signaling toward ERK/MAPK pathways while suppressing calcium flux, highlighting their context-dependent roles in cancer metastasis and autoimmune responses (Saotome et al., 2025).

## 3 Signal pathways of CXCR3

Most chemokine receptors transduce cellular signals through heterotrimeric GTP-binding proteins (G proteins) composed of α, β, and γ subunits. Binding of a chemokine to its receptor results in the activation of the α subunit and the subsequent dissociation of the βγ subunit complex (Berchiche and Sakmar, 2016).

Upon activation of CXCR3, the G protein regulates a wide range of signaling pathways, including adenylate cyclase, phospholipase C (PLC), phosphoinositide 3-kinase (PI3K), and mitogen-activated protein kinase (MAPK), which influence various cellular responses such as calcium influx, proliferation, integrin activation, actin remodeling, and migration (Smith et al., 2017).

**FIGURE 2:**
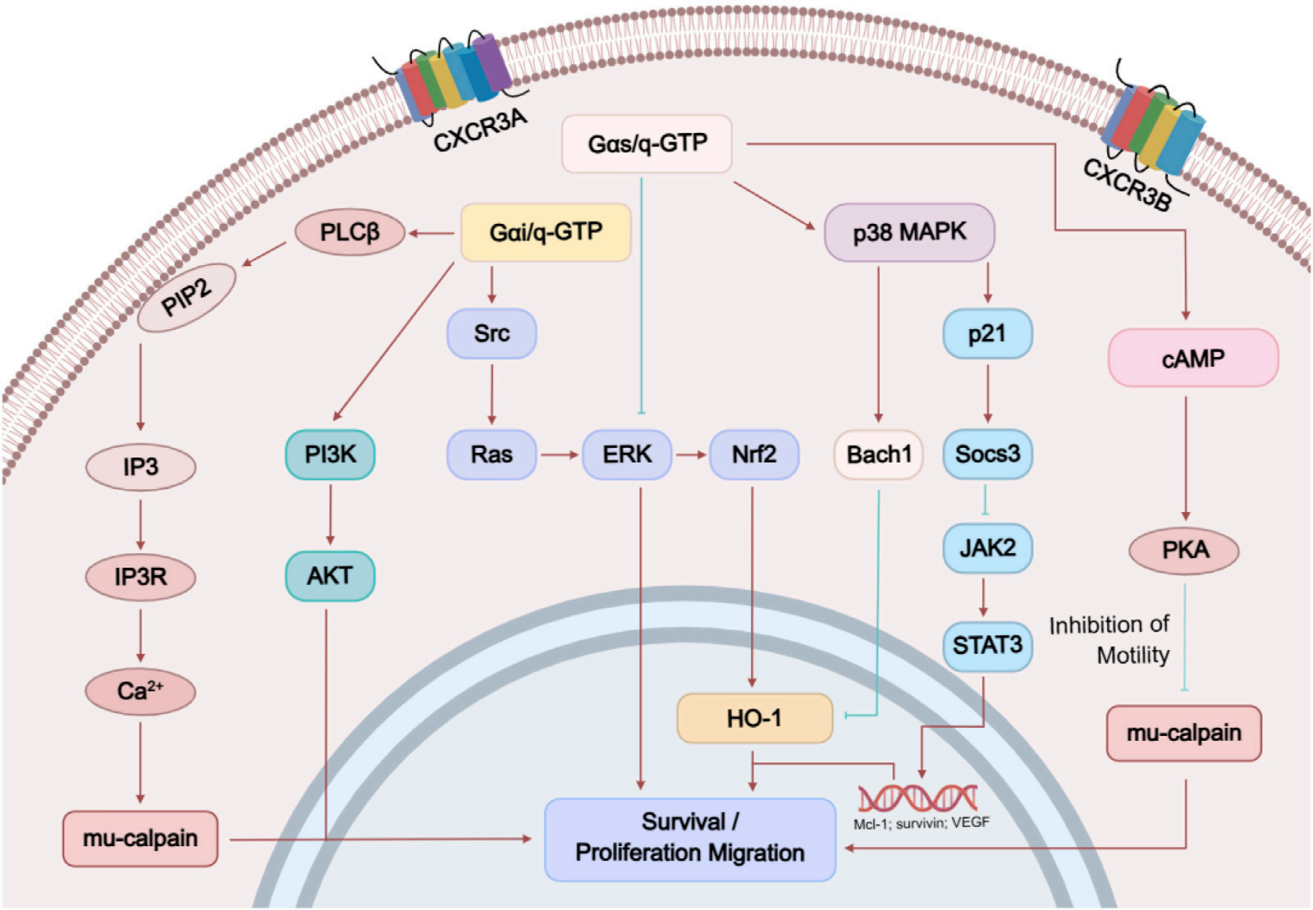
CXCR3 signal pathways.

The two distinct receptor subtypes of CXCR3, CXCR3-A and CXCR3-B, can activate different downstream signaling pathways (Figure 2). Both receptors can activate phospholipase Cβ (PLCβ), which hydrolyzes phosphatidylinositol bisphosphate (PIP2) to release two products: inositol trisphosphate (IP3, a general calcium ion influx second messenger) and diacylglycerol (DAG, a PKC activator). IP3 induces the downstream flow of Ca^2+^ into the cell, thereby activating u-calpain to weaken cell–substrate adhesion and promote cell motility; activated PKC enhances the activation of extracellular signal-regulated kinase (ERK), and the phosphorylation and activation of m-calpain (Billottet et al., 2013). In summary, CXCR3 signaling promotes cell migration via PLCβ activity. Additionally, CXCR3-B uniquely induces the accumulation of cAMP and activates protein kinase A (PKA, a cAMP-dependent kinase), which in turn inhibits the activation of m-calpain, blocking the release of signaling and suppressing cell migration.

Moreover, studies have shown that the different subtypes of CXCR3 may also activate distinct signaling pathways:

1. Gαi Activation: CXCR3-A can activate Gαi through CXCL10 and CXCL11, which is manifested as inhibition of forskolin-induced cAMP production. CXCR3-B only shows Gαi activation at a concentration of 100 nM CXCL11. It is noteworthy that CXCR3-B does not exhibit activation of classical G proteins (Gαi/o/q) under physiological ligand concentrations (1–10 nM CXCL11) (D’Uonnolo et al., 2022). Its N-terminal extended structure restricts conformational changes in the transmembrane domain, thereby hindering G protein coupling (Saha et al., 2025). However, CXCR3-B can still activate ERK signaling through a β-arrestin-dependent pathway and mediate chemokine internalization (Saha et al., 2025). This signaling bias may account for the unique role of CXCR3-B in tumor angiogenesis inhibition and provides a theoretical basis for the development of biased ligands (Zheng et al., 2022). CXCR3-Alt, however, fails to activate Gαi, even in the presence of any tested chemokine.
2. β-Arrestin: Both CXCR3-A and CXCR3-B exhibit ligand-dependent and ligand-independent interactions with β-arrestin1 and β-arrestin2. In the absence of a ligand, CXCR3-A shows significant basal BRET (bioluminescence resonance energy transfer) signals with both β-arrestins, which are further increased after CXCL11 stimulation. CXCR3-B exhibits a preference for β-arrestin2, observed both in the presence and absence of ligand. CXCR3-Alt does not show any ligand-dependent or independent interactions with β-arrestin1 or β-arrestin2.
3. Receptor Internalization: All CXCR3 variants undergo internalization in response to CXCL11, CXCL10, CXCL9, and CXCL4. CXCL4, in particular, induces strong receptor internalization in all three variants.
4. ERK1/2 Phosphorylation: CXCR3-A strongly induces ERK1/2 phosphorylation through CXCL11, CXCL10, and CXCL9, while the response to CXCL4 is weaker. CXCR3-B induces weaker ERK1/2 phosphorylation in response to CXCL11 and CXCL9, while CXCL10 and CXCL4 fail to induce significant responses. CXCR3-Alt shows limited ERK1/2 phosphorylation upon stimulation with CXCL11, CXCL10, and CXCL9.
5. Biased Agonism: Different splice variants of CXCR3 activate distinct signaling pathways, indicating the complexity of CXCR3 signaling. For example, CXCL11 and CXCL10 are full agonists for CXCR3-A, capable of activating both Gαi and ERK1/2 phosphorylation, while CXCL9 only shows a weaker response in ERK1/2 phosphorylation.
6. PTX Sensitivity: Studies also observed that β-arrestin recruitment to CXCR3-A and CXCR3-B is insensitive to PTX (pertussis toxin) treatment, suggesting that these responses may occur independently of Gαi signaling.

These findings highlight the distinct signaling properties of the various CXCR3 splice variants. They offer new insights into the role of CXCR3 in different biological processes and may aid in the development of targeted therapeutic strategies for specific CXCR3 variants.

## 4 The role of CXCR3 in the occurrence and development of diseases

**FIGURE 3:**
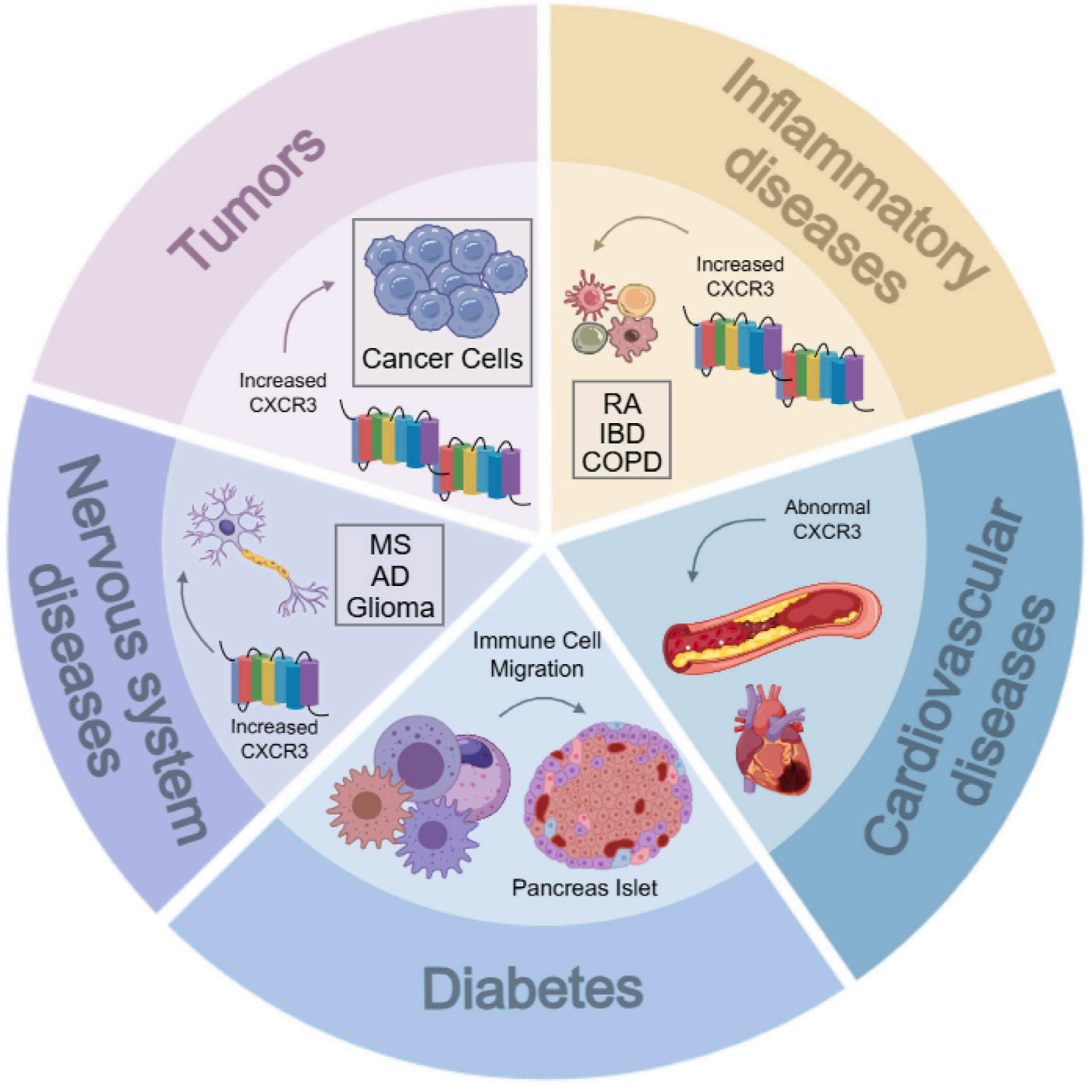
Relationship between CXCR3 and various diseases. RA, rheumatoid arthritis; IBD, inflammatory bowel diseases; COPD, chronic obstructive pulmonary diseases; MS, multiple sclerosis; AD, alzheimer’s diseases.

CXCR3 is directly or indirectly involved in tumor progression by regulating tumor growth, migration, invasion, angiogenesis, and immunity. CXCR3 is upregulated in many primary and metastatic tumors, including prostate, breast, colorectal, lung, ovarian, kidney, melanoma, and multiple myeloma. CXCR3 and one or more of its ligands have been found to be overexpressed in a variety of inflammatory diseases, including allograft rejection, atherosclerosis, autoimmune diseases, Examples include rheumatoid arthritis (RA), inflammatory bowel disease (IBD), chronic obstructive pulmonary disease (COPD), multiple sclerosis (MS), and systemic lupus erythematosus (SLE) (Figure 3).

### 4.1 Tumors

CXCR3 signaling is associated with tumor progression. CXCR3-A promotes mesenchymal cell migration, while CXCR3-B may inhibit movement. The expression of CXCR3-B regulates and is regulated by E-cadherin expression. Overexpression of CXCR3-A decreases E-cadherin, whereas overexpression of CXCR3-B leads to its upregulation. Both E-cadherin and CXCR3-B play roles in tumor suppression, but during early tumor metastasis, E-cadherin contributes to cell dormancy and survival. CXCR3-B may be involved in the early adaptation phase of metastasis, although the exact mechanisms remain unclear. Studies have also shown that E-cadherin expression is regulated by various mechanisms, including DNA methylation, transcriptional repression, and signal-induced unstable internalization. CXCR3-B may maintain the stability of adhesion complexes by inhibiting vinculin phosphorylation, though this hypothesis requires further validation. Furthermore, these findings may not be applicable to all cancer types, as methylation of E-cadherin may be the primary mechanism for its downregulation in other cancers. CXCR3 is a key chemokine receptor for Treg cell accumulation and immune suppression in tumors. Targeting CXCR3 could enhance anti-tumor immune responses, offering a new strategy for cancer treatment (Moreno Ayala et al., 2023).

#### 4.1.1 Hepatocellular carcinoma (HCC)

A study on tumor recurrence after liver transplantation found that the increase in circulating endothelial progenitor cells and CXCL10 was associated with a higher rate of tumor recurrence in patients who received small-volume liver transplants (Ling et al., 2014). In contrast, the recurrence rate was significantly lower in CXCL10-and CXCR3 knockout (KO) animals in a liver transplantation-like model. Importantly, in an orthotopic nude mouse model (Ding et al., 2016), injecting CXCL10 into the portal vein increased the number of pulmonary metastatic tumors. The authors of this study suggested that CXCL10 administration enhanced angiogenesis within tumors, leading to increased metastatic spread. However, this finding contradicts the classical understanding of CXCR3 function, as tumors in CXCL10-treated mice exhibited more CD34-positive cells, but these regions appeared to be smaller compared to untreated mice. Moreover, mice injected with endothelial progenitor cells exhibited larger tumors and multiple lesions in the liver, but did not show an increase in lung metastasis. Due to these findings, the involvement of angiogenesis in metastasis observed in this study is questionable and may require further investigation.

Other studies (Lan et al., 2014) have highlighted the role of CXCR3 signaling in HCC cells and its impact on metastasis. CXCR3 signaling, through the AKT/PI3K pathway and ERK1/2 signaling, plays a crucial role in promoting the invasive and migratory phenotypes of HCC cells. CXCR3 may influence HCC progression by promoting tumor cell proliferation and migration, as well as modulating the activity of immune cells within the tumor microenvironment. These findings suggest that CXCR3 could serve as a potential therapeutic target for HCC treatment (Ren et al., 2017).

#### 4.1.2 Lung cancer

In lung cancer, CXCR3 promotes tumor progression by regulating the expression levels of receptors on inflammatory cells or the expression levels of ligands on tumor cells (Gemelli et al., 2022). Unlike other cancers, in clinical specimens of non-small cell lung cancer (NSCLC), tumor cells and blood vessels are predominantly negative for CXCR3, while infiltrating immune cells show strong CXCR3 staining.

In NSCLC, the expression of CXCR3 and its ligands is elevated within the tumor microenvironment (TME), influencing the activity of natural killer (NK) cells and T cells, which are critical for the surveillance and elimination of cancer cells (Butler et al., 2017). The increased CXCR3 signaling may lead to the suppression of NK cell function, thereby promoting a “nurturing” phenotype that supports tumor growth and immune evasion. Additionally, CXCR3-positive NK cells may develop resistance to immune checkpoint inhibitor (ICI) therapy.

#### 4.1.3 Breast cancer

CXCR3 has been associated with poor survival outcomes in breast cancer patients. CXCR3-A signaling promotes breast cancer proliferation, while CXCR3-B may inhibit tumor growth. In the breast cancer cell lines MCF-7 and MDA-MB-231, the expression of CXCR3 splice variants A and B, as well as CTSB, is commonly observed. Upon binding to CXCR3, the chemokine ligands CXCL9 and CXCL10 can trigger the upregulation of CTSB, while the CXCR3-B specific ligand CXCL4 does not induce this response, suggesting that CXCR3-A is involved in the regulation of CTSB (Kundu et al., 2019). In breast cancer, the expression of CXCR3 is closely linked to tumor metastasis and prognosis. For example, high CXCR3 expression is associated with poor prognosis in breast cancer patients, and its expression level correlates positively with tumor size and the number of metastatic lymph nodes. Certain studies (Bronger et al., 2017) showed that CXCR3B expression is significantly upregulated in the breast cancer stem cell (CSC) subpopulation, and CXCL11 and CXCL10 can directly induce CSCs, indicating that CXCR3B may be involved in the characteristics of breast cancer stem cells and the metastatic spread of the tumor. Furthermore, CXCR3 expression in breast cancer is also linked to the tumor’s immune microenvironment (Cannon et al., 2021). In CXCR3-deficient mouse models, breast cancer progression is accelerated, which is associated with increased M2 polarization of macrophages, suggesting that CXCR3 may play a suppressive role in regulating the tumor immune microenvironment. Therefore, CXCR3 not only participates in the initiation and progression of breast cancer but may also regulate tumor metastasis and prognosis by influencing the tumor immune microenvironment. Its expression levels and function may vary depending on the tumor microenvironment, providing new potential therapeutic targets for breast cancer treatment.

#### 4.1.4 Prostate cancer (PC)

Prostate cancer (PC) is a global male urological disease known for its high incidence and mortality, particularly challenging to diagnose once bone metastasis occurs. The chemokine receptor CXCR3 plays a critical role in tumor angiogenesis and cell migration. Certain studies (Shen and Cao, 2015) used the PC-3 cell line model and gene silencing techniques to specifically downregulate two CXCR3 isoforms, CXCR3-A and CXCR3-B, confirming that downregulation of CXCR3-A led to an upregulation of CXCR3-B in PC-3 cells, further inhibiting cell proliferation and invasion. Furthermore, research by Wu et al. (2012) showed that CXCR3B is the major splice variant in normal human prostate tissues and epithelial cells, and that CXCL10 upregulates cAMP through CXCR3B, which in turn weakens m-calpain activity, thereby inhibiting the migration and invasion of RWPE-1 endothelial cells.

These findings provide a rationale for targeting CXCR3 as a potential therapeutic strategy to inhibit prostate cancer metastasis and progression. However, further experimental studies are needed to clarify the specific mechanistic role of CXCR3 in prostate cancer (Cannon et al., 2021).

#### 4.1.5 Renal cancer

The ratio of CXCR3-A to CXCR3-B is higher in renal cell carcinoma (RCC) tissues compared to normal kidney tissues, and the total expression of CXCR3 and CXCR3-A is significantly higher in metastatic RCC tissues than in non-metastatic RCC tissues (Gudowska-Sawczuk et al., 2020). A study by Sun et al. (2005) demonstrated that the angiogenesis-inhibiting protein IP-10 could be released by various mammalian cells through transduction with retroviral vectors carrying the human IP-10 gene (RCR vectors). These cells, cultured *in vitro* for at least 3 months, indicated that gene therapy based on RCR vectors could provide a long-term supply of anti-angiogenesis proteins during tumor treatment, thus being more effective and convenient for inhibiting tumor growth. The expression of CXCR3 and its ligands on monocytes was found to be elevated in metastatic renal cancer patients treated with high-dose IL-2, exerting some anti-angiogenic effects (Reckamp et al., 2007). Datta et al. (2010) confirmed via flow cytometry that the high expression of CXCR3-B in the human renal cancer cell line Caki-1 promoted apoptosis. Using protein chip technology and small interfering RNA (siRNA), they discovered that high expression of CXCR3-B significantly downregulated the expression of heme oxygenase-1 (HO-1), an anti-apoptotic protein, and that inhibiting HO-1 expression enhanced renal cancer cell apoptosis. These findings help explain the possible mechanism by which CXCR3-B inhibits renal cancer cell growth.

#### 4.1.6 Colorectal cancer

18%–34% of colorectal cancer (CRC) patient samples show strong CXCR3 staining, and most of these CXCR3-positive patients are also diagnosed with lymph node metastasis (Abron et al., 2017). Patients expressing CXCR3 have a poorer prognosis compared to those who do not express CXCR3 or express CXCR4 or CCR7. Previous studies have demonstrated high expression of cytotoxic CXCR3 ligands on CD8^+^ T cells in colorectal cancer tissues. CXCR3 and its ligands exhibit differential expression at inflammatory sites and within tumors. Moreover, the expression of CXCR3 and its ligands correlates with the presence of effector T cells in tumor tissues and with patients’ disease-free survival (Jin et al., 2018).

One of the leading causes of mortality in CRCis invasion and metastasis. The cooperative interaction between CXCR3 and CXCR4 plays a crucial role in regulating CRC cell invasion. Recent studies (Cannon et al., 2021) have found that CRC cells expressing higher levels of CXCR3 and CXCR4 exhibit increased invasiveness. CXCR3 enhances CXCR4 functionality in CRC cells by forming heterodimers at the cell surface and preventing CXCR4 internalization. Taken together, targeting CXCR3 may represent a promising strategy for the clinical treatment of CRC cell invasion and metastasis.

#### 4.1.7 Other cancers

CXCR3-A expression is elevated in ovarian cancer and endometriosis tissues compared to normal ovarian tissue, while CXCR3-alt and CXCR3-B show upregulation and downregulation, respectively, in ovarian cancer tissues (Cannon et al., 2021). CXCR3-A is predominantly expressed on cancer cells and infiltrating lymphocytes, whereas CXCR3-B and CXCR3-alt are expressed in microvessels. The expression of CXCL4 and CXCL4L1 in ovarian cancer associated with endometriosis is significantly lower than in endometriosis alone (Windmüller et al., 2017). CXCR3-positive tumor cells are strongly associated with immune reactivity in tumors with thickness greater than 1 mm or with invasive, lethal melanoma (Jenkins et al., 2015). n multiple myeloma, CXCR3 expression increases across different pathological stages, with higher expression observed in stage III compared to stage I (Ma et al., 2015). The presence of both CXCR3-A and CXCR3-B subtypes is associated with the regulation of the cell cycle and apoptosis.

Glioblastoma multiforme (GBM), a highly invasive brain tumor, has limited treatment options and poor prognosis (Chan et al., 2023). CXCR3, a chemokine receptor, functions with both autocrine and paracrine signaling mechanisms. Current clinical treatments targeting CXCR3 ligands, which are associated with the CXCR3 axis, have demonstrated antitumor effects in GBM (Guo and Gao, 2015).

### 4.2 Inflammatory diseases

CXCR3 plays a crucial role in various inflammatory diseases. Karkada et al. demonstrated the key role of CXCR3 in T cell migration to inflamed joints and the progression of arthritis in an adjuvant-induced arthritis model in Lewis rats. The study found that the use of anti-CXCR3 monoclonal antibodies significantly reduced T cell migration and alleviated the severity of arthritis, including reductions in clinical symptoms, weight loss, and neutrophil accumulation in the joints. These findings suggest that blocking CXCR3 could offer a novel therapeutic strategy for rheumatoid arthritis (RA). Furthermore, serum CXCL4 has been identified as a general biomarker for inflammatory bowel disease (IBD). IFN-γ-induced chemokines may contribute to the progression of IBD and exacerbate the associated gastrointestinal inflammation (Meuwis et al., 2007).

The inflammation in chronic obstructive pulmonary disease (COPD) involves both innate and adaptive immunity. CXCR3, through binding with its specific ligands, plays an important role in regulating tissue inflammation and damage. In acute animal models subjected to cigarette smoke (CS) or pathogen challenges, CXCR3 knockout (KO) alleviated lung inflammation and pathology (Li et al., 2021).

### 4.3 Neurodegenerative diseases

CXCR3 plays a significant role in neurodegenerative diseases. CXCR3-positive T cells are increased in the blood of patients with multiple sclerosis (MS), and its ligand CXCL10 is expressed by astrocytes in MS brain lesions, suggesting that CXCR3 may be involved in the immune response and lesion development in MS (Zhou et al., 2019). The expression of CXCR3 is elevated in glioma cells and is associated with tumor malignancy. CXCR3 antagonists have been shown to inhibit glioma growth and prolong survival. Additionally, CXCR3 expression in neurons and neural processes in Alzheimer’s disease (AD) patients is correlated with AD-related pathological changes. CXCR3-deficient mice exhibit improved spatial memory performance. CXCR3 antagonists have demonstrated therapeutic effects in various neurodegenerative disease models, indicating that CXCR3 may represent a potential therapeutic target for these disorders (Müller et al., 2010).

### 4.4 Diabetes

CXCR3 is a chemokine receptor expressed on activated T cells and natural killer (NK) cells. By binding to ligands such as CXCL10, it promotes the migration of immune cells to the pancreatic β cells, exacerbating the immune attack on these cells and playing a critical role in the development of type 1 diabetes (T1D). The activation of this receptor also stimulates the production of cytokines like interferon-γ, further enhancing the immune response and inflammation. Moreover, CXCR3 is involved in the development of insulitis, affecting the function and survival of β cells, as β cells themselves can produce CXCL10, which in turn inhibits their proliferation. Certain viral infections can also induce β cells to produce CXCL10, attracting immune cells and exacerbating inflammation. Thus, CXCR3 not only promotes autoimmune attacks on pancreatic β cells but may also contribute to disease progression by influencing cytokine production and β cell self-regulation. This makes CXCR3 a potential therapeutic target, and blocking CXCR3 may slow or prevent the development of type 1 diabetes (Antonelli et al., 2014).

### 4.5 Cardiovascular diseases

CXCR3 plays a key role in cardiovascular diseases, such as atherosclerosis, cardiac hypertrophy, heart failure, cardiac transplant rejection, and myocardial infarction, through its interaction with various ligands. In atherosclerosis, CXCR3 and its ligands influence plaque formation and rupture. In cardiac hypertrophy and heart failure, elevated levels of CXCR3 ligands are associated with disease progression. In cardiac transplantation, increased expression of CXCR3 ligands is linked to transplant rejection, while in myocardial infarction, the recruitment of CXCR3-positive cells is critical for cardiac repair (Altara et al., 2018). Additionally, CXCR3 and its ligands are involved in other cardiovascular conditions such as hypertension and myocarditis, indicating that they not only play a significant role in the onset and progression of these diseases, but also may serve as therapeutic targets, offering potential new strategies for the treatment of cardiovascular diseases (Satarkar and Patra, 2022).

## 5 CXCR3 small molecular inhibitors

In mouse disease models, antibodies or small molecule antagonists of CXCR3 have been shown to significantly delay the progression of diseases such as atherosclerosis, transplant rejection, and cancer, making CXCR3 antagonists a key focus in drug discovery. Several classes of small molecule CXCR3 antagonists have been developed, three of which have demonstrated efficacy in preclinical models, though only two have progressed to clinical trials (Yuan, 2024). These antagonists include piperazine-based piperidine compounds (e.g., SCH 546738) and 8-aziquinazoline derivatives (e.g., AMG487). The diversity of CXCR3 antagonists complicates the establishment of a universal pharmacophore model, and newly emerging ligands do not always contain highly basic groups. Structural and spatial differences between small molecule antagonists and chemokines may lead to distinct binding sites, such as the binding site of TAK-779 with CCR5 [130]. Antagonists with different structures may act as inverse agonists on active mutants of CXCR3, providing new directions for future drug design (Ahmad et al., 2023).

### 5.1 Specific antagonists

#### 5.1.1 Piperazine-based piperidine compounds

**FIGURE 4:**
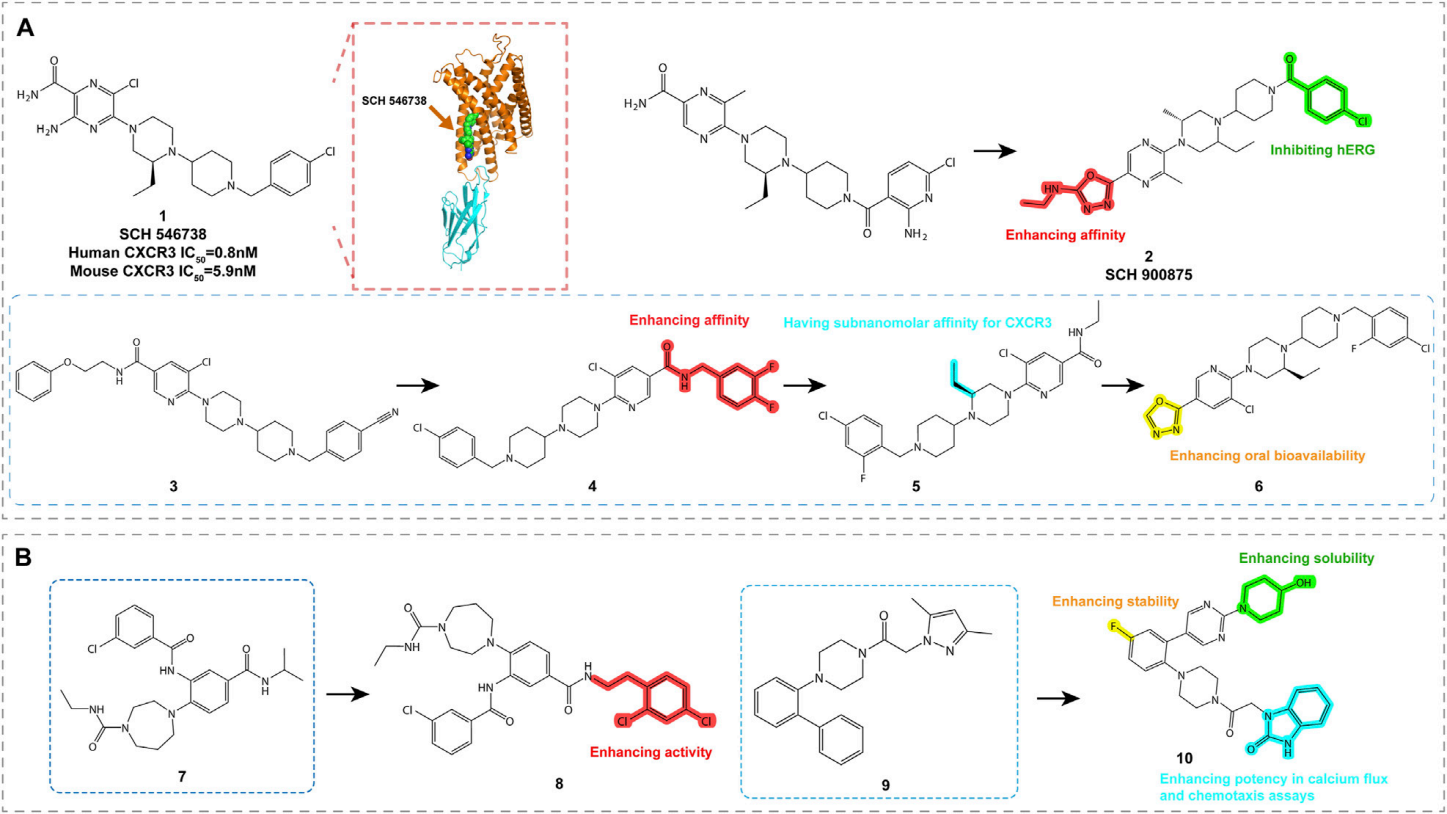
Structures and Optimization Pathways of Piperazine-based Piperidine Compounds. **(A)** Structure and optimization of piperazine-based piperidine compounds. For compound **3**, the chloro-benzyl group is a key moiety for enhancing the compound’s affinity. While shortening the N-benzylamide group decreases its affinity, introducing small alkyl groups, particularly (S)-substituents on the piperazine ring, significantly improves its *in vitro* activity. Halogenation of the benzamide group in compound 4 strengthens its binding affinity to CXCR3, and substituting the amide group with heterocyclic moieties increases its oral bioavailability. **(B)** Structure and optimization of arylpiperazine compounds. Compounds **7** and **8** contain amide or aryl groups at the ortho position of homopiperazine, which is crucial for maintaining their activity. SAR studies of compound **9** indicate that the central phenyl ring can accommodate a third substituent, such as an amide group, similar to compounds studied by Ligand Pharmaceuticals, or a halogen atom, and can also be modified to include a pyridine ring. The central phenyl ring of compound **10** can tolerate additional substituents, such as halogens or amide groups, which provide opportunities to fine-tune the pharmacological properties of the compound.

Ligands based on piperazine-piperidine scaffolds were identified as CXCR3 antagonists between 2006 and 2009, leading to the development of compounds 1 through 6, as shown in Figure 4A.

SCH 546738 (Compound **1**, Table 2) is a piperazine-piperidine-based CXCR3 antagonist with a high affinity of 0.4 nM. It effectively non-competitively displaces radiolabeled CXCL10 and CXCL11 (IC_50_ = 10 nM) and significantly inhibits CXCR3-mediated chemotaxis in activated T cells. In autoimmune disease models in mice and rats, compound **1** alleviated disease symptoms and demonstrated favorable pharmacokinetic properties (Poulet et al., 2010). These findings suggest that compound **1** has potential as a therapeutic agent for autoimmune diseases and the prevention of transplant rejection. However, despite its positive effects in preclinical models, compound **1** requires further optimization before clinical trials to ensure its safety and efficacy in humans, as well as to explore potential combinations with other therapeutic modalities (Jenh et al., 2012).

**TABLE 2:** Summary of CXCR3 specific antagonists.

Structural class	Compound	Disease focus	Study setting	PK/PD characteristics	Key results
**Piperazine-based Piperidine**	SCH 546738 (**1**)	Autoimmune diseases, transplant rejection	*In vivo*	High affinity (0.4 nM), non-competitive ligand displacement (IC50 = 10 nM), favorable PK in rodents	Inhibiting T-cell chemotaxis; alleviating autoimmune symptoms; requiring safety/efficacy optimization for clinical trials
	SCH 900875 (**2**)	Autoimmune diseases (arthritis, EAE)	*In vivo*	High affinity, improved metabolic stability, moderate oral bioavailability	Effective in collagen-induced arthritis and EAE models; species-specific side effects in animals
	Compound **3**	General CXCR3-related diseases	*In vitro*	High affinity (pKi = 7.0), pIC50 values: 7.3 (CXCL9), 7.7 (CXCL10), 7.1 (CXCL11)	Chlorobenzyl group critical for affinity; low oral bioavailability requires optimization
	Compound 4	General CXCR3-related diseases^a^	*In vitro*	High affinity (pKi = 7.4), poor oral PK (AUC = 0.92 μM h), significant hERG inhibition	Enhancing binding but poor metabolic stability; cardiac toxicity concerns
	Compound **5**	General CXCR3-related diseases	*In vitro*	Extremely high affinity (pKi = 9.5), poor oral bioavailability (AUC = 0.95 μM h)	Improving affinity via ethyl substitution, but PK remains suboptimal
	Compound **6**	General CXCR3-related diseases	*In vitro*	Improved oral bioavailability (AUC = 1.8 μM h), high affinity (pKi = 8.9), high hERG inhibition	Heterocyclic substituents enhanced PK; cardiac toxicity unresolved
**Arylpiperazines**	Compound **7**	Inflammatory/Autoimmune	*In vitro*	Amide group at ortho position; CXCR3 antagonistic activity	Foundational activity but lacks optimization
	Compound **8**	Inflammatory/Autoimmune	*In vitro*	Aryl group at ortho position; high activity (pIC50 = 7.2), selectivity over 14 GPCRs	Optimized version of Compound 7; improving activity and selectivity
	Compound **9**	Inflammatory/Autoimmune	*In vitro*	Submicromolar activity; low metabolic stability (human liver microsomes), high permeability (PAMPA)	Demonstrating permeability but requiring metabolic stability optimization
	Compound **10**	Inflammatory/Autoimmune	*In vitro*	Improved solubility and bioavailability; pIC50 = 8.0 (calcium flux), 7.7 (chemotaxis)	Enhancing PK properties compared to Compound 9; remaining unstable in human liver microsomes
**Azaquinazolinone**	Compound **11**	Inflammatory diseases	*In vitro*/*in vivo*	Submicromolar activity, low oral bioavailability in rats	Initial lead; foundational for optimization
	AMG 487 (**12**)	Psoriasis, inflammatory diseases	Phase II clinical trial	Time-dependent CYP3A4 inhibition (via M4 metabolite), variable human exposure	Failed Phase II due to inconsistent PK; inhibited CXCR3-mediated migration
	Compound **13**	Inflammatory diseases	*In vitro*/*in vivo*	Improved stability and bioavailability, avoided CYP3A4 inhibition	Potent CXCR3 antagonism; advanced from PK optimization
	Compound **14**	CXCR3-related diseases	*In vivo*	Good oral bioavailability, efficacy in preclinical models	Promising candidate for CXCR3-related diseases
	Compound **15**	Transplant rejection	*In vivo*	Prolonged graft survival, reduced IFN-γ-producing T cells	Synergistic with anti-CD154 mAbs; potential for immunoregulation
	Compound **16**	Inflammatory diseases	*In vitro*/*in vivo*	Strong affinity (IC50 = 1.0 nM), low clearance, good oral bioavailability	Inhibiting bleomycin-induced inflammation; lower plasma concentration vs AMG487
	Compound **17**	Inflammatory diseases	*In vitro*/*in vivo*	High affinity (IC50 = 0.9 nM), low clearance, moderate oral bioavailability	Effective in pulmonary inflammation models; low drug-drug interaction risk
	Compound **18**	CXCR3-related diseases	*In vitro*	High CXCR3 affinity, inverse agonistic effects	Inhibiting constitutive receptor signaling; novel mechanism
	Compound **19**	Autoimmune/inflammatory diseases	*In vitro*/*in vivo*	High affinity (IC50 = 12 nM), low clearance, reduced metabolic risk	Improving solubility/stability; reducing glutathione binding for safety
	Compound **20**	Autoimmune/inflammatory diseases	*In vitro*/*in vivo*	High affinity (IC50 = 7.8 nM), low clearance, reduced metabolic risk	Improving solubility/stability; reducing glutathione binding for safety
	JN-2 (**21**)	Breast cancer bone metastasis	*In vitro*/*in vivo*	Inhibiting CXCL10/CXCR3/NF-κB axis	Reducing breast cancer cell motility and bone destruction
	NBI-74330 (**22**)	Rheumatoid arthritis	*In vitro*	Binding activity to CXCR3, unresolved PK/safety issues	Tool compound for design insights; not advanced clinically
**Arylsulfonamide**	Compound **23**	CXCR3-related diseases	*In vitro*	Sub-micromolar activity; optimized to amide (pIC50 = 7.9), poor stability	Structural optimization improved activity; intermediates enhanced stability
	Compound **24**	CXCR3-related diseases	*In vivo*	Favorable PK in mice; improved stability via isoelectronic analogs	Promising PK profile but lacking further development data
	Compound **25**	CXCR3-related diseases	*In vitro*	High activity (IC50 = 13 nM in chemotaxis), poor metabolic stability	Key SAR insights: N-substituent (R3) and sulfonamide ring (R1) critical; tetrazole derivatives improved stability/solubility. Not clinically viable but a lead for future optimization
**Spiropiperidine**	Compound **26**	Inflammatory/Immune-related diseases	*In vitro*	pIC50 = 6.9 (human CXCR3 calcium flux assay); limited *in vivo* PK/PD data	High antagonistic potency; SAR insights for future design
**Benzimidazole**	Compound **27**	Inflammatory diseases	*In vitro*	Sub-micromolar affinity; substituents critical for affinity/solubility	Identified key SAR; foundational for optimization
	Compound **28**	Inflammatory diseases	*In vitro*	IC50 = 80 nM; good oral bioavailability, limited stability in rat liver microsomes	Functional antagonist; improving PK but metabolic instability
	Compound **29**	Inflammatory diseases	*In vitro*	Higher affinity (human/mouse CXCR3); metabolically unstable	Effective functional antagonist; guiding further optimization
**Phenylethylpiperidine Derivatives**	Compound **30**	Inflammatory diseases	*In vitro*	Most active in HTS; enantiomer-specific muscarinic receptor activity	SAR revealed optimal aromatic ring substitution; retaining activity
	Compound **31**	Inflammatory diseases	*In vitro*	Derived from SAR of Compound 30; selectivity undisclosed	Structural insights for CXCR3 antagonist design
**1-Phenyl-3-piperidin-4-ylurea Derivatives**	Compound **32**	Inflammatory diseases	*In vitro*	High lipophilicity; prioritized for analog synthesis	Key lead molecule; enabling rapid structural optimization
	Compound **33**	Inflammatory diseases	*In vitro*	IC50 = 16 nM; improved solubility, *in vitro* stability, low CYP inhibition	Promising drug template with balanced PK/PD.
	Compound **34**	Inflammatory diseases	*In vitro*/*in vivo*	Antagonistic activity in CXCR3 internalization assays; good *in vivo* characteristics	Validated in receptor internalization models; potential for clinical translation
	Compound **35**	Inflammatory diseases	*In vivo*	Good PK in mice; complete inhibition of CXCR3 internalization	Advanced candidate with robust *in vivo* efficacy

^a^ CXCR3-related diseases: Includes cancers, inflammatory diseases, autoimmune disorders, neurodegenerative conditions, diabetes, cardiovascular diseases, and transplant rejection.

Through structure-activity relationship (SAR) studies and chemical modifications, including changes to the benzyl group (Nair et al., 2014) and optimization of halogenated benzamide derivatives, SCH 900875 (compound **2**, Table 2) was developed. Compound **2** exhibits high affinity and good *in vitro* activity. In animal models, compound **2** effectively treats various autoimmune diseases, including collagen-induced arthritis and experimental autoimmune encephalomyelitis (EAE). Despite showing potential side effects in animal models, possibly due to alterations in the membrane lipid composition of red blood cells and platelets, its effects in humans may vary due to species differences.

Compound **3** (Table 2) was identified through high-throughput screening and demonstrated high affinity in radioligand binding assays (pKi = 7.0). It effectively antagonizes the binding of CXCL9, CXCL10, and CXCL11 to CXCR3, with pIC50 values of 7.3, 7.7, and 7.1, respectively (McGuinness et al., 2009; Shao et al., 2010; Kim et al., 2011; Nair et al., 2014). SAR studies of this compound indicated that the chlorobenzyl group is critical for its high affinity. Despite its strong *in vitro* activity, the oral bioavailability of compound **3** needs further improvement. These findings provide important insights for the further optimization of CXCR3 antagonists.

Through halogenated benzylamide substitution on compound **3**, compound **4** (Table 2) was developed, which exhibited high affinity (pKi = 7.4), but poor pharmacokinetics upon oral administration (AUC = 0.92 μM h) (Shao et al., 2010). Although structural modification enhanced binding to CXCR3, metabolic stability was not improved, and the compound exhibited significant hERG inhibition. Further shortening the benzylamide group to an ethyl group resulted in compound **5** (Table 2), which displayed extremely high affinity (pKi = 9.5) *in vitro*, but no improvement in oral bioavailability (AUC = 0.95 μM h) (Mladic et al., 2015). Compound 6 (Table 2), achieved by replacing the amide group with heterocyclic substituents, improved both oral bioavailability (AUC = 1.8 μM h) and affinity (pKi = 8.9), but still exhibited high hERG inhibitory activity (Kim et al., 2011). These compounds provide promising candidates for the treatment of CXCR3-related diseases, although further optimization is required to balance metabolic stability and cardiac toxicity.

#### 5.1.2 Arylpiperazines

Aryl piperazine compounds and their derivatives have been developed as CXCR3 antagonists, with these compounds featuring amide or aryl groups at the ortho position of the piperazine ring. High-throughput screening identified compounds **7** and **8** (Figure 4B; Table 2), which belong to the arylpiperazine class and contain either an amide or aryl group. Compound **7** demonstrated CXCR3 antagonistic activity, while compound **8**, as an optimized version, showed high activity in in vitro assays (pIC50 = 7.2) and exhibited good selectivity over 14 other GPCRs (Cole et al., 2006; Afantitis et al., 2009).

Compound **9** (Figure 4B; Table 2), an aryl piperazine derivative discovered by Boehringer Ingelheim, exhibited submicromolar activity. Although its stability in human liver microsomes was low, it demonstrated high permeability in PAMPA (parallel artificial membrane permeability assay) screening (Prokopowicz et al., 2024). Compound **10** (Table 2), an improved version of compound **9** (Figure 4B), exhibited better solubility and bioavailability, as well as good potency in calcium flux and chemotaxis assays (pIC50 values of 8.0 and 7.7, respectively). These characteristics make compound 10 a promising candidate for CXCR3 antagonism research, despite its lower stability in human liver microsomes (Gottschling et al., 2009).

#### 5.1.3 Azaquinazolinone compounds

**FIGURE 5:**
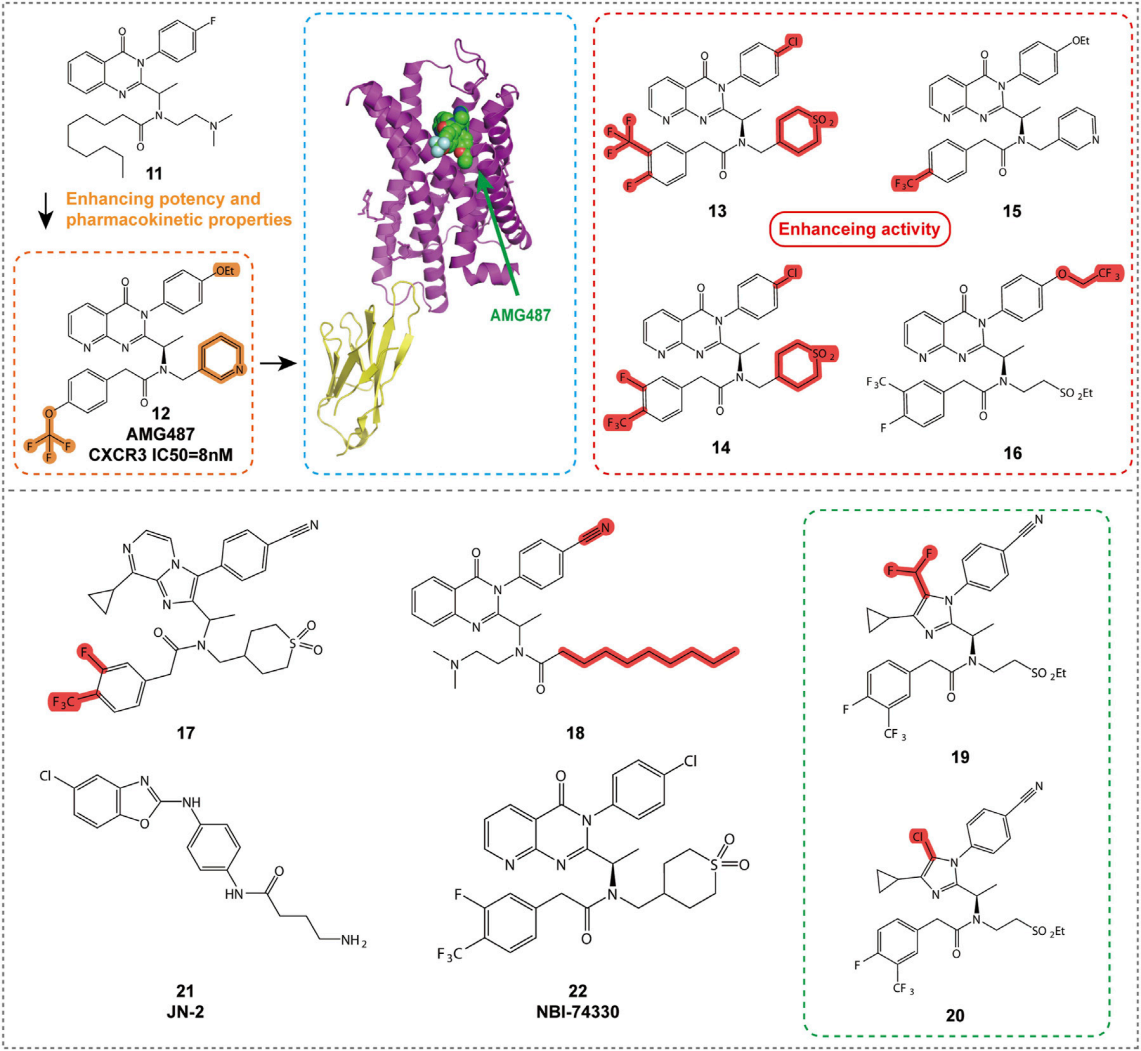
Structures and Optimization Pathways of 8-Azaquinazolinone Compounds.

Quinazolinone-based compounds are a significant area of research for CXCR3 antagonists, with compounds **11**, **12**, and **13** (Figure 5, Table 2) discovered and optimized by ChemoCentryx and Amgen. These 8-azaquinazolinone compounds were identified through high-throughput screening and optimized for CXCR3 receptor antagonism.

Compound **11** was the initial active compound identified through high-throughput screening, exhibiting submicromolar activity and effectively blocking the binding of CXCR3 to its ligands. Although its oral bioavailability in rats was low, its discovery provided a starting point for further optimization (Johnson et al., 2007). After several rounds of optimization and efficacy validation in a bleomycin-induced murine model of pulmonary cell recruitment, 8-nitroquinazolinone, AMG487 (compound **12**), was selected as the clinical candidate. It demonstrated inhibition of CXCR3-mediated cell migration in both *in vitro* and animal models, showing potential for the treatment of various inflammatory diseases (Tonn et al., 2009). Further studies revealed that the M2 phenolic metabolite, M4, played a key role in the time-dependent inhibition (TDI) of CYP3A4, covalently binding to the Cys239 residue of CYP3A4 and leading to enzyme inactivation. Additionally, M4 was further metabolized to form M5, and these complex metabolic pathways collectively influenced the human pharmacokinetics of compound **12** (Henne et al., 2012). However, despite promising results in preclinical models, compound 12 failed to demonstrate efficacy in a Phase IIa clinical trial for psoriasis, possibly due to its time-dependent pharmacokinetic characteristics leading to variable exposure. As a result, further development of the compound was discontinued (Horuk, 2009).

To address the pharmacokinetic issues associated with compound **12**, researchers optimized compounds containing ethylsulfonyl groups and explored new core structures to improve stability. This led to the discovery of cyanide-substituted compounds that effectively avoided CYP3A4 inhibition, resulting in the development of compounds **13** and **14** (Figure 5; Table 2) (Chen et al., 2012). Compound 13 exhibited potent CXCR3 antagonistic activity in in vitro assays and, through structural optimization, demonstrated improved *in vivo* stability and bioavailability. Compound 14, on the other hand, showed good oral bioavailability and efficacy in preclinical models, supporting its potential as a candidate for the treatment of CXCR3-related diseases.

Compound **15** (Figure 5; Table 2), a new non-peptide small molecule CXCR3 antagonist, prolonged the survival time of allogeneic hearts in a mouse heart transplant model, potentially offering therapeutic effects in preventing acute transplant rejection (Rosenblum et al., 2009). Moreover, compound **15** reduced the frequency of interferon-γ-producing donor-reactive T cells, and when used in combination with anti-CD154 monoclonal antibodies, significantly extended graft survival. These findings provide new insights into the role of CXCR3 in transplant immunology and offer a scientific basis for the development of novel immunoregulatory therapeutic strategies. Future research should further investigate the mechanism of action of compound **15** and its potential application in clinical transplantation, particularly regarding its efficacy and safety when used in combination with other immunosuppressive agents. Additionally, compound **15** was shown to inhibit CXCL9, CXCL10, and CXCL11-induced cell migration *in vitro*, disrupt the development of donor-specific responses and IFN-γ production, and prolong graft survival. These results suggest that CXCR3 may serve as a viable therapeutic target for preventing acute organ rejection.

Compound **16** (Figure 5; Table 2) demonstrates excellent pharmacological properties and favorable pharmacokinetic characteristics. *In vitro* studies show strong affinity for the CXCR3 receptor (IC50 = 1.0 nM) and excellent inhibition of cell migration (IC50 = 0.8 nM) (Liu et al., 2009). In multiple species, compound **16** exhibits low to moderate clearance rates and good oral bioavailability. It effectively inhibits bleomycin-induced cell infiltration and shows similar inhibitory effects to AMG 487, but with lower plasma concentrations.

Compound **17**, with an imidazopyrazine core structure, demonstrates high affinity *in vitro* (IC50 = 0.9 nM) and strong inhibition of cell migration (IC50 = 18.9 nM). In pharmacokinetic studies in rats, compound **17** (Figure 5; Table 2) shows low clearance and moderate oral bioavailability, and it exhibits significant *in vivo* efficacy in a mouse model of pulmonary inflammation. It also presents a low risk of drug-drug interactions, making it a strong candidate for the treatment of various inflammatory diseases (Du et al., 2009).

Compound **18** (Figure 5; Table 2) is a derivative of 3-phenyl-3H-quinazolin-4-one, exhibiting the highest CXCR3 receptor affinity among its class. It effectively blocks CXCR3-mediated calcium ion release, demonstrating moderate inhibitory activity (Ki = VUF5834). As a constitutively active mutant of compound **18**, it shows complete inverse agonistic effects, inhibiting constitutive receptor signaling. This opens up new directions for the treatment of CXCR3-related diseases (Storelli et al., 2005).

Compounds **19** and **20** are imidazole derivatives, optimized to show high pharmacological activity and favorable pharmacokinetic properties. By introducing substituents on the imidazole ring, these compounds enhance affinity for the CXCR3 receptor and improve *in vivo* stability and solubility, while reducing metabolic activation risks. *In vitro* assays demonstrate that compounds **19** and **20** (Figure 5; Table 2) possess high affinity (IC50 values of 12 nM and 7.8 nM, respectively) and effectively inhibit CXCR3-mediated cell migration (Du et al., 2008). Pharmacokinetic studies show low clearance in rats, moderate mean residence time, and good oral bioavailability, making them potential candidates for oral administration. The structural modifications of compounds **19** and **20** also reduce binding to glutathione, decreasing the risk of metabolic activation and improving drug safety. These compounds exhibit excellent characteristics both *in vitro* and *in vivo*, making them ideal tools for studying the role of CXCR3 in disease and valuable candidates for the development of new therapies for autoimmune and inflammatory diseases.

CXCL10 and its receptor CXCR3 play a crucial role in breast cancer bone metastasis and osteoclast activation. Researchers developed the CXCR3 antagonist JN-2 (compound **21**, Figure 5; Table 2), which was found to inhibit CXCL10 expression and the motility of breast cancer cells, reducing bone destruction. These findings reveal the role of the CXCL10/CXCR3/NF-κB signaling pathway in breast cancer progression, providing a potential target for new therapeutic strategies (Kim et al., 2018; Jin et al., 2017).

Compound **22** (NBI-74330, Figure 5; Table 2), although showing binding activity to the CXCR3 receptor, was not advanced to clinical trials due to pharmacokinetic properties or potential side effects. Nevertheless, the research on this neutral 8-azaquinazolinone derivative provides important insights for the design of CXCR3 antagonists and may represent a new therapy for rheumatoid arthritis (Ahmad et al., 2023).

The introduction of small molecular substituents at the adjacent nitrogen atom on the left side of the core structure significantly enhances the CXCR3 antagonistic activity of compound **17**. Compound **19**, which contains a difluoromethyl substituent at position 5 of the imidazole ring, and compound **20**, which contains a chlorine substituent, both reduce binding to glutathione (GSH), thereby decreasing the risk of metabolic activation and improving the safety profile of the drugs. Compound **22** primarily occupies the minor binding site of the receptor, located around transmembrane regions (TMs) 2, 3, and 7.

#### 5.1.4 Arylsulfonamide compounds

**FIGURE 6:**
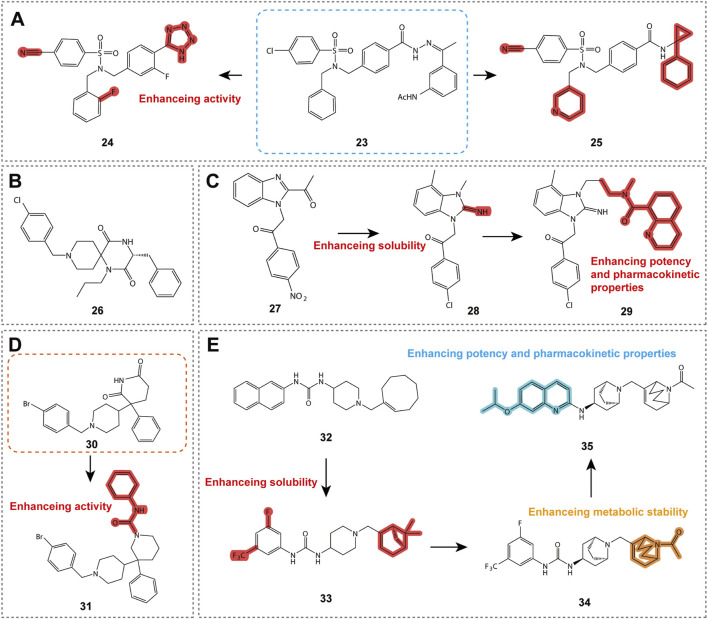
Structures and optimization pathways of aryl sulfonamide compounds, spiropiperidine compounds, benzimidazole derivatives, phenylethylpiperidine derivatives, and 1-phenyl-3-piperidin-4-ylurea derivatives. **(A)** Structure and optimization of aryl sulfonamide compounds. Replacing the acetohydrazone structure of compound **23** with an amide does not affect its CXCR3 antagonistic activity. In the design of compound **25**, a diversity exploration was conducted on the amide N-substituent R3, revealing a preference for more hydrophobic groups, particularly thiophene methylene and 1-phenyl-cycloprop-1-yl groups. Additionally, compound **25** contains a crucial chlorine atom at the R1 position on the sulfonamide ring, which is essential for maintaining the activity of the compound. Furthermore, a second benzyl substituent R2 on the sulfonamide nitrogen also shows some tolerance, with a preference for aromatic or heteroaromatic groups containing an unsubstituted methylene chain, especially the 2-pyridyl group. **(B)** Spiropiperidine compound structure. The different cyclic structures on the piperidine ring of compound **26** may be related to its potential binding to the CXCR3 receptor. **(C)** Structure and optimization of benzimidazole compounds. SAR studies show that the small aliphatic substituent at the C-4 position of compound **27** enhances its potency, leading to the discovery of compound **28**. **(D)** Structure and optimization of phenylethylpiperidine derivatives. **(E)** Structure and optimization of 1-phenyl-3-piperidin-4-ylurea derivatives. For compound **34**, acylated piperidine exhibits superior CXCR3 activity compared to the corresponding piperidine.

Compounds **23** and **24** (Figure 6A; Table 2) are CXCR3 antagonists discovered by Merck Serono, belonging to the aryloxy sulfonamide class of derivatives (Crosignani et al., 2010). During screening of 90,000 compounds, compound **23** exhibited sub-micromolar activity, and its structure was optimized into an amide without affecting activity, achieving a pIC50 value of 7.9 for the optimized compound. Although these compounds have relatively poor stability, intermediate acids and their isoelectronic analogs (such as tetrazole) exhibited improved stability and activity. Compound **24** showed favorable pharmacokinetic properties in mice, although further studies have not been reported to date.

Compound **25** (Table 2) represents another important CXCR3 antagonist discovery, with an IC50 value of 13 nM in chemotaxis assays, demonstrating excellent activity. This compound has specific requirements for the position of the amide N-substituent (R3) and the sulfonamide ring (R1), while the R2 position prefers aromatic or heteroaromatic groups with an unsubstituted methylene chain. Although compound **25** exhibits good receptor activity, its metabolic stability is poor. The research team employed various strategies to improve its metabolic stability, resulting in tetrazole derivatives with enhanced stability and solubility. Compound **25** provides valuable SAR insights for the design of CXCR3 antagonists. Although it may not be suitable for direct clinical use, it offers a promising starting point for the discovery of new CXCR3 antagonists.

#### 5.1.5 Spiropiperidine compounds

As shown in Figure 6B, compound **26** (Table 2) is a CXCR3 antagonist developed by Ono Pharmaceutical Company, belonging to the spiropiperidine class of compounds (Habashita and Shibayama, 2011). This compound exhibits a pIC50 value of 6.9 in human CXCR3 calcium flux assays, indicating a high level of antagonistic potency. Although the *in vivo* pharmacodynamics and pharmacokinetic properties of these compounds have not been extensively reported, the discovery of compound **26** provides valuable SAR insights for the design of CXCR3 antagonists, which may aid in the development of drugs for the treatment of inflammatory and immune-related diseases in the future.

#### 5.1.6 Benzimidazole compounds

Benzimidazole compounds are shown in Figure 6C. Compound **27**, (Table 2) discovered by Abbott Laboratories, is a CXCR3 antagonist and belongs to the benzimidazole derivative class, exhibiting sub-micromolar affinity. SAR studies reveal that certain substituents on the benzimidazole core are critical for maintaining both affinity and solubility (Hayes et al., 2008a). Compound **28** (Table 2), a derivative of compound **27**, serves as a functional antagonist with an IC50 of 80 nM, demonstrating good oral bioavailability and half-life, but its stability in rat liver microsomes is limited (Hayes et al., 2008b). Further optimization led to the identification of compound **29** (Table 2), which exhibits higher affinity for both human and mouse CXCR3 and is an effective functional antagonist, although it remains metabolically unstable. These findings provide valuable information for the design of CXCR3 antagonists and the research of treatments for related inflammatory diseases (Hayes et al., 2008b).

#### 5.1.7 Phenylethylpiperidine derivatives

Compounds **30** and **31** (Table 2) are CXCR3 antagonists discovered by Johnson & Johnson through high-throughput screening in CXCR3-transfected Chinese hamster ovary (CHO) cells, belonging to the phenylethylpiperidine derivative class, as shown in Figure 6D (Pierre Bongartz et al., 2008). After screening 256,000 compounds, these compounds exhibited activity against CXCR3, with compound **30** showing the most significant activity. Both enantiomers of compound **30** demonstrated CXCR3 inhibitory activity, but only one exhibited muscarinic receptor activity. SAR studies revealed the optimal substitution pattern for the two aromatic rings and indicated that the oxazine ring, when devoid of a carbonyl group, could retain activity as long as the nitrogen atom was substituted with an acyl or sulfonyl group. Compound **31** is a derivative derived from these conditions. Although compounds **30** and **31** (Table 2) exhibited CXCR3 activity and selectivity, other characteristics have not been disclosed. These compounds provide valuable structural insights for the drug design and development of CXCR3 antagonists, contributing to future research on the treatment of CXCR3-related inflammatory diseases.

#### 5.1.8 1-Phenyl-3-piperidin-4-ylurea derivatives

Compound **32** (Figure 6E; Table 2) is a CXCR3 antagonist discovered by UCB after screening 15,000 compounds, representing a key molecule within a series of compounds (Allen et al., 2007). Despite its high lipophilicity, compound **32** was selected for further study due to its molecular properties and potential for rapid analog synthesis. The improved compound **33** (Figure 6E; Table 2) demonstrated high activity (IC50 = 16 nM), along with improved physicochemical properties, good *in vitro* stability, and low CYP inhibition, making it a promising template for drug development (Watson et al., 2007). Further research led to the discovery of compound **34** (Figure 6E; Table 2), which exhibited good characteristics both *in vitro* and *in vivo*, and demonstrated antagonistic activity in CXCR3 receptor internalization assays *in vivo*. The latest findings include compound **35** (Table 2), which showed good pharmacokinetic properties in mice and completely inhibited CXCR3 internalization following administration. These compounds provide valuable information for the drug design of CXCR3 antagonists and the study of treatments for related inflammatory diseases.

### 5.2 Non-Specific Antagonists (broad-spectrum antagonists)

#### 5.2.1 Quaternary ammonium derivatives

**FIGURE 7:**
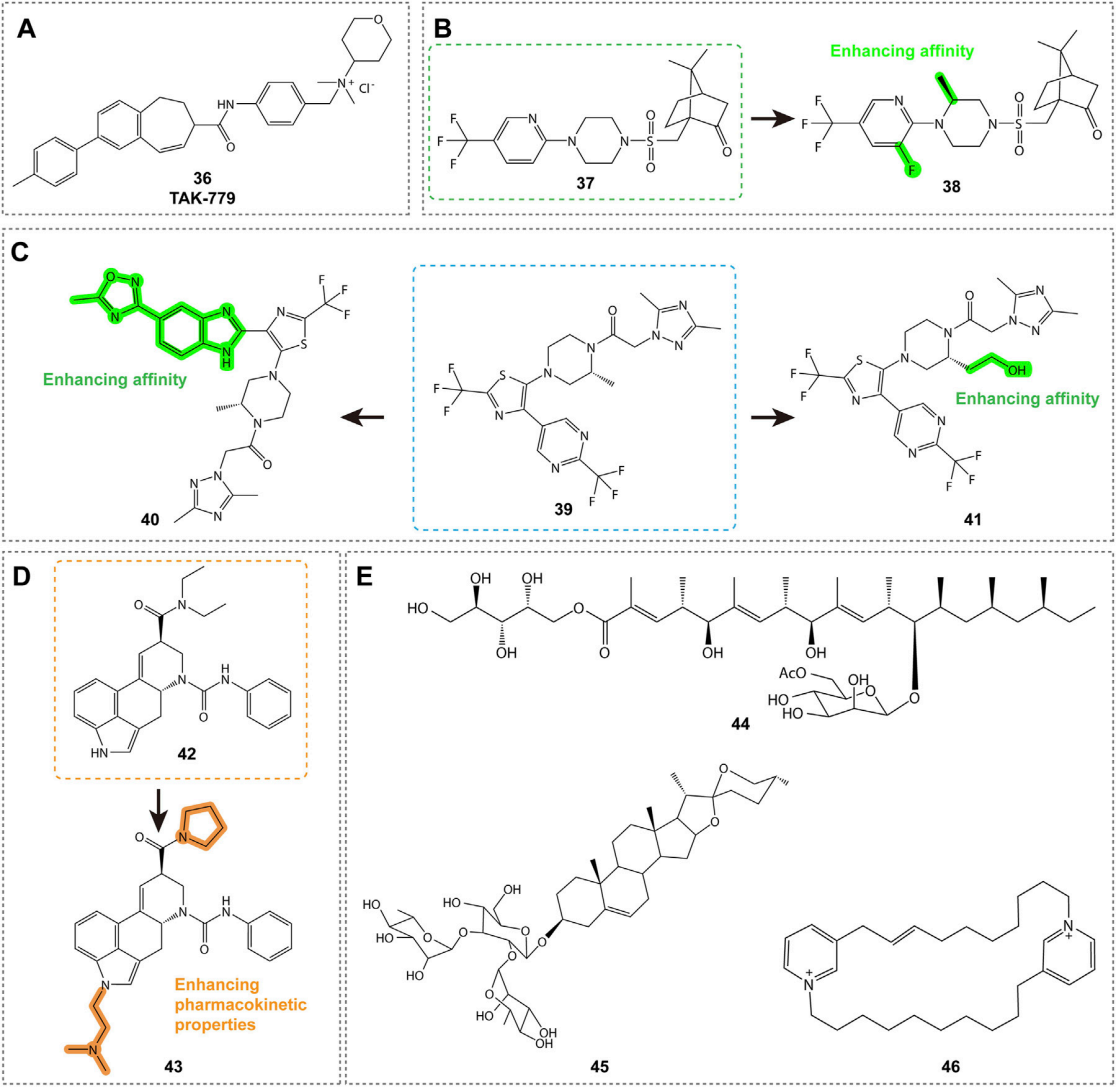
Structures and Optimization Pathways of Non-Specific Antagonists and Natural Products. **(A)** Quaternary Ammonium Salt Derivatives: The binding site of compound **36** to CXCR3 differs from that of another antagonist, VUF10085, in that it does not rely on the key amino acid residues that are essential for VUF10085 binding. **(B)** Camphor Sulfonamide Compounds: The structure and optimization of camphor sulfonamide derivatives. Compound **38** was discovered by systematically modifying the phenyl ring, piperidine ring, and camphor bicyclic structure of compound **37**, incorporating optimal substituents. **(C)** Benzimidazole Derivatives: Structure and optimization of benzimidazole-based CXCR3 antagonists. **(D-E)** Natural Products: Structure and optimization pathway of natural products. For compound **42**, replacing the N,N-diethylamide group with a pyrrolidinamide resulted in a tenfold increase in activity. Introducing hydrophobic substituents on the indole nitrogen of compound **42** led to reduced activity, while incorporating polar substituents not only maintained activity in binding and cell assays but also enhanced activity in whole blood experiments.

As shown in Figure 7A, TAK-779 (compound **36**, Table 3) is a broad-spectrum small-molecule chemokine receptor antagonist that antagonizes CXCR3, CCR5, and CCR2b. Initially developed as a CCR5 antagonist to inhibit HIV-1 entry, TAK-779 also antagonizes CCR2 and CXCR3, demonstrating potential for treating a range of Th1 cell-mediated diseases such as rheumatoid arthritis and cancer (Nedjai et al., 2015). In a rat small intestine transplantation model, TAK-779 significantly prolonged graft survival, reduced the number of infiltrating cells at the transplant site, and decreased the expression of CCR5 and CXCR3. Additionally, it reduced the total number of T cells involved in transplant rejection (Xu et al., 2008). Furthermore, TAK-779 was shown to reduce the migration of inflammatory cells to the central nervous system, decreasing the incidence and severity of experimental autoimmune encephalomyelitis, without affecting T cell function (Ni et al., 2009). When used in combination with FK506, TAK-779 significantly prolonged allogeneic small intestine graft survival, with TAK-779 inhibiting T cell migration to the graft by blocking CCR5 and CXCR3 (Takama et al., 2011). These characteristics make TAK-779 a promising therapeutic strategy.

**TABLE 3:** Summary of CXCR3 non-specific antagonists (broad-spectrum antagonists).

Structural class	Compound	Disease focus	Study setting	PK/PD characteristics	Key results
**Quaternary Ammonium Derivatives**	TAK-779 (**36**)	HIV-1 entry; Th1 cell-mediated diseases (rheumatoid arthritis, cancer); transplant rejection; autoimmune encephalomyelitis	*In vitro*/*in vivo*	Not explicitly described	Prolonging graft survival; reducing infiltrating cells and CCR5/CXCR3 expression; suppressing T cell migration; reducing EAE severity; synergy with FK506
**Camphor Sulfonamide Derivatives**	Compound **37**	Th1 cell-mediated diseases (implied via CXCR3 antagonism)	*In vitro*	Not described	Initial hit from HTS; structural precursor to compound 38
	Compound **38**	Th1 cell-mediated diseases	*In vivo*	Low solubility; short half-life; low bioavailability	Moderate CXCR3 antagonism; good selectivity; unfavorable PK in rats; potential for structural optimization
**Benzimidazole Derivatives**	ACT-660602 (**39**)	Lung inflammation	*In vitro*	Improved metabolic stability; low hERG inhibition; primarily metabolized by CYP2D6 (polymorphic)	Reduced CXCR3+ CD8^+^ T cell recruitment; halted due to variable plasma exposure from CYP2D6 metabolism
	ACT-672125 (**40**)	Lung inflammation	*In vivo*	Not described	Inhibited CXCR3+ T cell recruitment
	ACT-777991 (**41**)	Recent-onset type 1 diabetes	*In vivo*/*in vitro*	Excellent safety profile; favorable pharmacokinetics	Dose-dependent efficacy and target engagement; progressed to Phase I with strong safety profile

#### 5.2.2 Camphor sulfonamide derivatives

As shown in Figure 7B, compounds **37** and **38** (Table 3) are CXCR3 antagonists discovered by GlaxoSmithKline, with compound **37** being a camphor sulfonamide derivative identified through high-throughput screening (Wang et al., 2009). Compound **38** is an optimized version of compound **37**, showing moderate CXCR3 antagonistic activity and good selectivity, but exhibiting unfavorable pharmacokinetic properties in rats, including low solubility, short half-life, and low bioavailability. While there is potential for improving these properties through structural modifications, specific details have not yet been disclosed.

#### 5.2.3 Benzimidazole derivatives

Compound **39** (ACT-660602, Figure 7C; Table 3) is a CXCR3 antagonist with high biological activity, effectively inhibiting cell migration while improving metabolic stability and cardiac safety (low hERG channel inhibition) (Meyer et al., 2022). In a mouse lung inflammation model, a 30 mg/kg dose of ACT-660602 significantly reduced the recruitment of CXCR3+ CD8^+^ T cells. Despite favorable results from *in vitro* cardiac safety and toxicity assessments, the preclinical development of compound **39** was halted due to its primary metabolism via the polymorphic cytochrome P450 enzyme 2D6, which can lead to highly variable plasma exposure.

CXCR3 antagonists **40** (ACT-672125) and 41 (ACT-777991) were shown to inhibit the recruitment of CXCR3+ T cells in a lung inflammation model (Figure 7C; Table 3) (Caroff et al., 2022). Notably, compound 41 has progressed to Phase I clinical trials as a clinical candidate for the treatment of recent-onset type 1 diabetes. It demonstrated dose-dependent efficacy and target engagement, with excellent characteristics and safety profile.

### 5.3 Natural products

Natural products are shown in Figures 7D-E. Compounds **42** and **43** (Table 4) are promising CXCR3 antagonists discovered by Novartis (Thoma et al., 2009; Rolf et al., 2006). Compound **42** is an ergot alkaloid derivative with high selectivity, showing no binding to other GPCRs, and demonstrating metabolic stability in human microsomes (Auberson et al., 2014). Compound **43** exhibits favorable pharmacokinetic properties and is a promising tool compound for exploring the role of CXCR3 in disease models (Thoma et al., 2011).

**TABLE 4:** Summary of CXCR3 natural products.

Structural class	Compound	Disease focus	Study setting	PK/PD characteristics	Key results
**Ergot Alkaloid Derivative**	Compound **42**	CXCR3-related diseases^a^	*In vitro*	High selectivity (no binding to other GPCRs); High metabolic stability	Highly selective CXCR3 antagonist with metabolic stability; potential tool compound for research
**Natural Product**	Compound **43**	CXCR3-related diseases	*In vivo*/*in vitro*	Favorable pharmacokinetic properties	Promising tool compound for investigating CXCR3’s role in disease models
**Natural Product**	Compound **44**	CXCR3-related diseases	*In vitro*	Not described	Exhibiting CXCR3 binding activity; providing novel insights for antagonist design and inflammatory disease research
**Diosgenin Glycoside**	Compound **45**	CXCR3-related diseases	*In vivo*/*in vitro*	Not described	Binding to CXCR3; supports development of CXCR3 antagonists and inflammatory disease studies
**Cyclic 3-Alkylpyridinium Salt**	Compound **46**	CXCR3-related diseases	*In vivo*/*in vitro*	Not described	Demonstrating CXCR3 binding activity; offering new directions for antagonist design and inflammation research

^a^ CXCR3-related diseases: Includes cancers, inflammatory diseases, autoimmune disorders, neurodegenerative conditions, diabetes, cardiovascular diseases, and transplant rejection.

Merck identified three potential CXCR3 antagonist molecules through screening 51,000 extracts: compound **44** (roselipins, Table 4) (Tomoda et al., 2003), compound **45** (a diosgenin glycoside, Table 4) (Watson et al., 2007), and compound **46** (a cyclic 3-alkylpyridinium salt natural product, Table 4) (Turk et al., 2008). These compounds exhibit binding activity to CXCR3, offering new directions for the design of CXCR3 antagonists and the study of related inflammatory diseases.

## 6 Conclusion

In recent decades, the role of CXCR3 and its ligands in oncology and inflammatory diseases has attracted extensive attention. CXCR3 not only plays a key role in the development and metastasis of various tumors, but it is also critically involved in the pathogenesis of inflammatory responses and autoimmune diseases. With a deeper understanding of the CXCR3 signaling pathway, small molecule antagonists targeting CXCR3 have emerged as a potential therapeutic strategy. Despite some success in preclinical models, the clinical application of CXCR3 antagonists still faces several challenges.

First, the selectivity and specificity of CXCR3 antagonists remain critical issues in drug development. Non-selective antagonists may interfere with other biological processes, leading to adverse effects. Therefore, developing highly selective CXCR3 antagonists is crucial to minimizing off-target effects and improving therapeutic efficacy. In addition, the pharmacokinetic and pharmacodynamic properties of the drugs are also important factors that determine their clinical application. Many promising candidate drugs fail in clinical trials due to poor pharmacokinetic properties or insufficient efficacy.

Looking to the future, the research on CXCR3 antagonists needs to progress in several key areas: 1) Target Validation and Disease Models: Further studies are needed to validate the role of CXCR3 in various diseases and to develop more relevant animal models and preclinical models that better mimic human diseases. 2) Drug Design and Optimization: Through structure-based drug design and computational chemistry methods, more precise small molecules can be designed to bind with the CXCR3 receptor, improving their affinity and selectivity. Additionally, optimizing the chemical structure of the drugs can improve their pharmacokinetic properties, enhance oral bioavailability, and address metabolic stability issues. 3) Combination Therapy: Considering the complex roles of CXCR3 in multiple cell types and signaling pathways, single-drug therapies may struggle to achieve optimal therapeutic outcomes. Therefore, exploring combination therapies involving CXCR3 antagonists and other drugs may enhance therapeutic efficacy, particularly in the treatment of tumors and autoimmune diseases.

Notably, recent preclinical studies have shown that β-arrestin-biased inhibitors such as ACT-777991 can specifically block the infiltration of CXCR3+ CD8^+^ T cells into the heart, significantly improving survival rates in models of immune checkpoint inhibitor-induced myocarditis (Wang et al., 2022). In contrast, Gαi-biased compounds may be more suitable for inhibiting tumor metastasis. Future efforts should focus on combining cryo-electron microscopy structures of the CXCR3-B isoform to design subtype-specific biased inhibitors (Rajagopal et al., 2013).

In conclusion, while the research and development of CXCR3 antagonists face challenges, the continued advancement of science and technology, along with a deeper understanding of CXCR3’s biological functions, may lead to more CXCR3 antagonists entering clinical application. This could offer new therapeutic options for patients with tumors and inflammatory diseases. The search for effective chemokine receptor candidates continues, and the concept of receptor allosteric targeting may be a promising direction for future research.
